# Supplementary figures and images for: Preprinting Microbiology
Source: mBio. 2017 May 23;8(3):e00438-17. doi: 10.1128/mBio.00438-17 (PMC5442452; doi:10.1128/mBio.00438-17)

**Supplemental Figure 1. Screen shot of the preprint for this manuscript at *bioRxiv.***


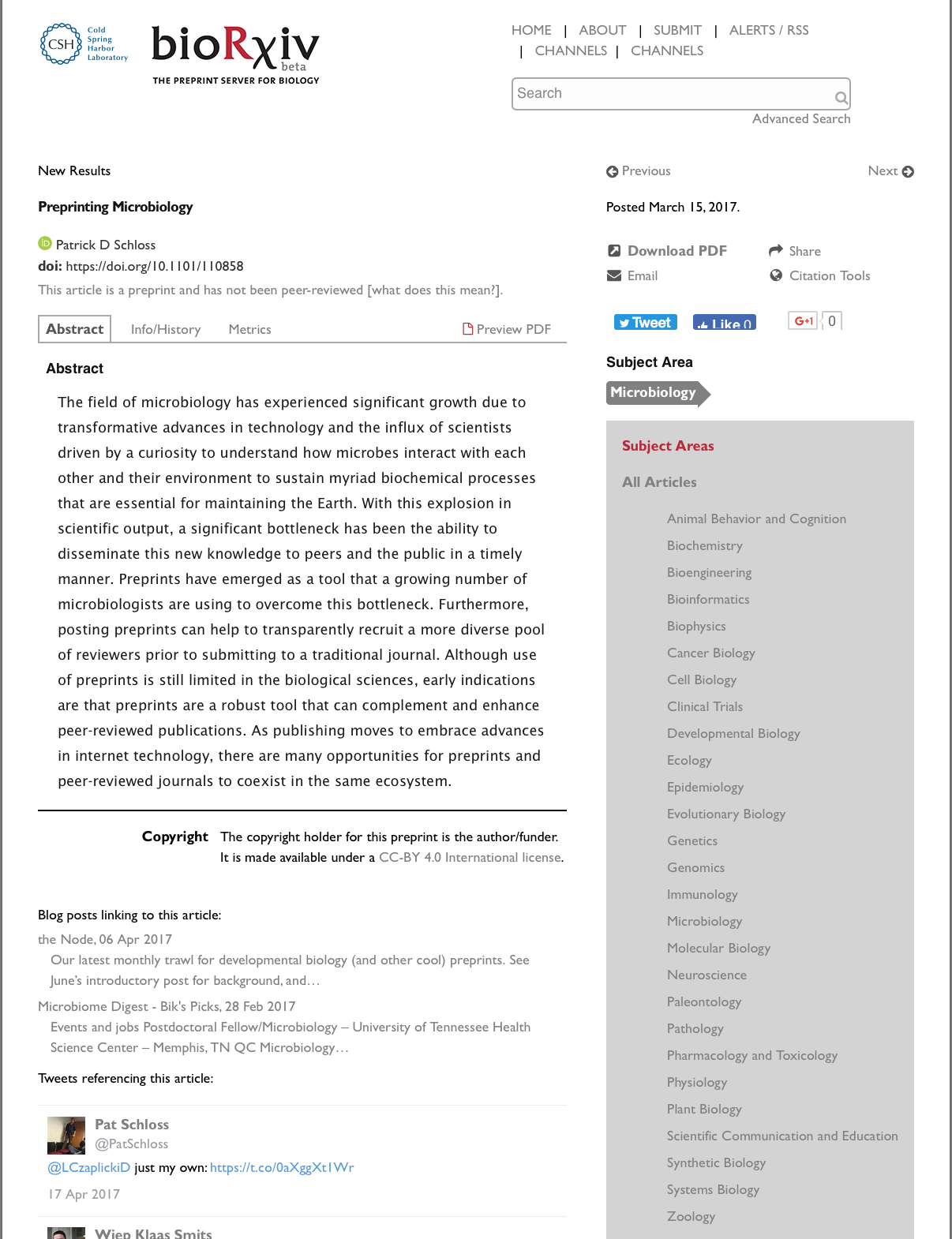

Supplement: FIG S1 [file mbo003173314sf1.docx]

**Supplemental Figure 2. Screen shot of a preprint by the author hosted at *PeerJ Preprints.***


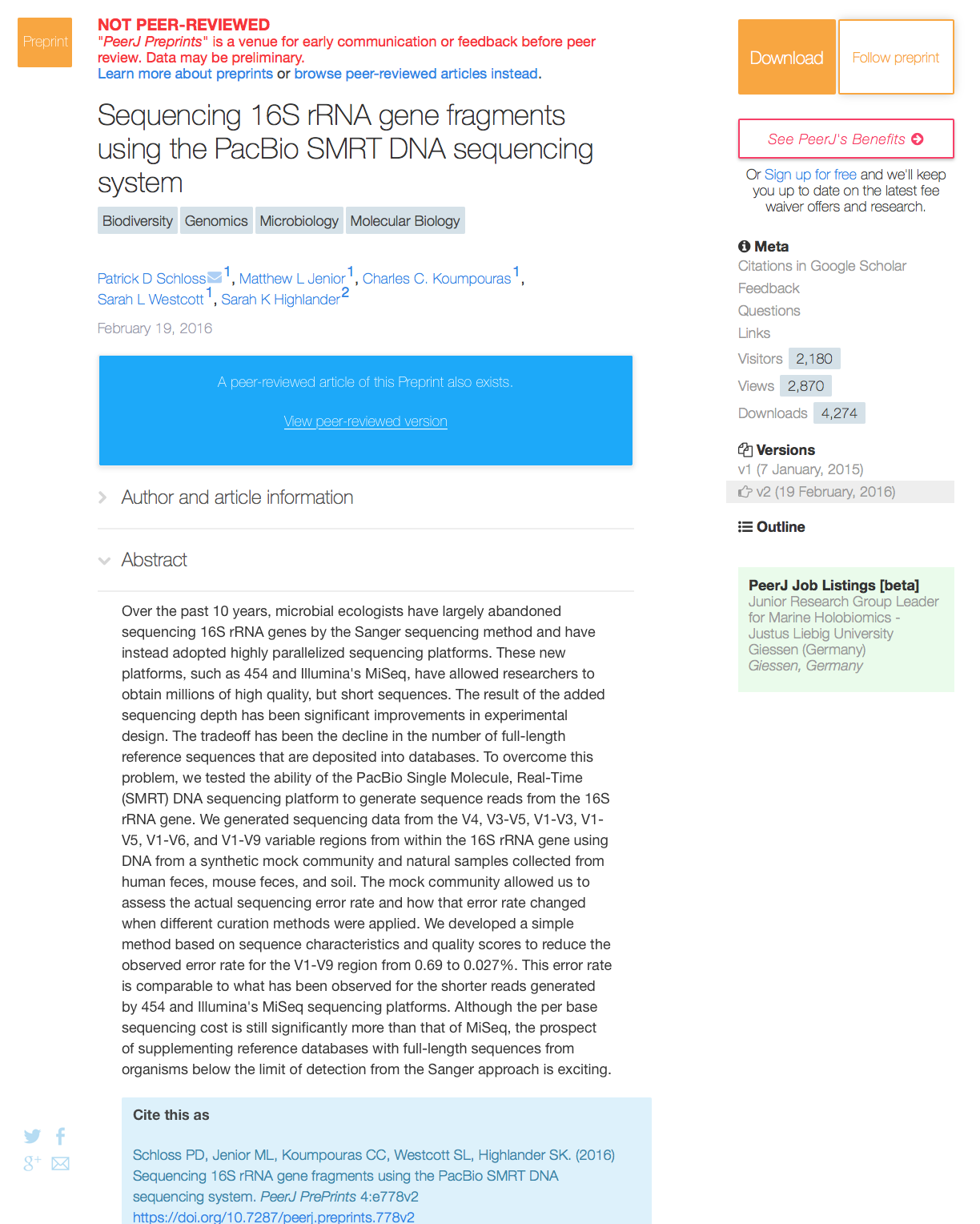

Supplement: FIG S2 [file mbo003173314sf2.docx]
